# Colorectal Cancer: A Review of Carcinogenesis, Global Epidemiology, Current Challenges, Risk Factors, Preventive and Treatment Strategies

**DOI:** 10.3390/cancers14071732

**Published:** 2022-03-29

**Authors:** Md. Sanower Hossain, Hidayah Karuniawati, Ammar Abdulrahman Jairoun, Zannat Urbi, Der Jiun Ooi, Akbar John, Ya Chee Lim, K. M. Kaderi Kibria, A.K. M. Mohiuddin, Long Chiau Ming, Khang Wen Goh, Muhammad Abdul Hadi

**Affiliations:** 1 Department of Biomedical Science, Kulliyyah of Allied Health Sciences, International Islamic University Malaysia, Kuantan 25200, Pahang, Malaysia; 2 Faculty of Science, Sristy College of Tangail, Tangail 1900, Bangladesh; 3 Discipline of Social and Administrative Pharmacy, School of Pharmaceutical Sciences, Universiti Sains Malaysia, Gelugor 11800, Pulau Pinang, Malaysia; hk170@ums.ac.id (H.K.); aajairoun@dm.gov.ae (A.A.J.); 4 Department of Pharmacology and Clinical Pharmacy, Faculty of Pharmacy, Universitas Muhammadiyah Surakarta, Surakarta 57102, Indonesia; 5 Health and Safety Department, Dubai Municipality, Dubai 67, United Arab Emirates; 6 Department of Industrial Biotechnology, Faculty of Industrial Sciences & Technology, Universiti Malaysia Pahang, Kuantan 26300, Pahang, Malaysia; urbi.zannat@gmail.com; 7 Department of Oral Biology & Biomedical Sciences, Faculty of Dentistry, MAHSA University, Jenjarom 42610, Selangor, Malaysia; djooi@mahsa.edu.my; 8 Institute of Oceanography and Maritime Studies (INOCEM), Kulliyyah of Science, International Islamic University Malaysia, Kuantan 25200, Pahang, Malaysia; akbarjohn@iium.edu.my; 9 PAP Rashidah Sa’adatul Bolkiah Institute of Health Sciences, Universiti Brunei Darussalam, Gadong BE1410, Brunei; yachee.lim@ubd.edu.bn; 10 Department of Biotechnology & Genetic Engineering, Mawlana Bhashani Science and Technology University, Tangail 1902, Bangladesh; km_kibria@mbstu.ac.bd (K.M.K.K.); mohiuddin@mbstu.ac.bd (A.K.M.M.); 11 Faculty of Data Science and Information Technology, INTI International University, Nilai 71800, Negeri Sembilan, Malaysia; khangwen.goh@newinti.edu.my; 12 College of Pharmacy, QU Health, Qatar University, Doha 2713, Qatar; mabdulhadi@qu.edu.qa

**Keywords:** anus cancer, colon cancer, drug resistance, prevalence, rectum cancer

## Abstract

**Simple Summary:**

The high lethality of colorectal cancer (CRC) is anticipated to continue in the following decades. Early-stage CRC is entirely treatable with surgery and adjuvant therapy (chemotherapy/radiotherapy). However, recurrence is common, and cancer drug resistance increases the chances of treatment failure. At the same time, there are many risk factors leading to high prevalence among both genders. Despite significant improvements in treating other cancers, CRC management is not yet at a satisfactory level. In this comprehensive review, we discussed the development of CRC and its associated risk factors, preventive and treatment strategies and proposed some recommendations. Furthermore, besides chemotherapy and targeted therapy, we discussed natural products as therapeutics for CRC.

**Abstract:**

Colorectal cancer (CRC) is the second most deadly cancer. Global incidence and mortality are likely to be increased in the coming decades. Although the deaths associated with CRC are very high in high-income countries, the incidence and fatalities related to CRC are growing in developing countries too. CRC detected early is entirely curable by surgery and subsequent medications. However, the recurrence rate is high, and cancer drug resistance increases the treatment failure rate. Access to early diagnosis and treatment of CRC for survival is somewhat possible in developed countries. However, these facilities are rarely available in developing countries. Highlighting the current status of CRC, its development, risk factors, and management is crucial in creating public awareness. Therefore, in this review, we have comprehensively discussed the current global epidemiology, drug resistance, challenges, risk factors, and preventive and treatment strategies of CRC. Additionally, there is a brief discussion on the CRC development pathways and recommendations for preventing and treating CRC.

## 1. Introduction

Colorectal cancer (CRC), which comprises colon and/or rectum cancer, represents a significant health problem as the world’s third most commonly diagnosed and second most fatal cancer globally [1]. Approximately 9.4% of cancer-related deaths were due to CRC in 2020 [2]. However, in light of the significant increase in the number of identified cases in the older population, it is estimated that the global incidence of CRC will more than double by 2035, with the most significant increase occurring in less developed nations [3].

CRC is a disorder that occurs exclusively in the colon or rectum and is caused by the colon’s aberrant proliferation of glandular epithelial cells. There are three principal types of CRC: Sporadic, hereditary, and colitis-associated. The number of CRC cases is increasing globally day by day. Both environmental and genetic factors determine the risk of developing CRC. In addition, the risk of developing CRC in patients with long-standing ulcerative colitis and Crohn’s disease increases with age [4]. Multiple studies have demonstrated that risk factors for CRC include diet and lifestyle, family history, and chronic inflammation [5].

However, the most effective method of preventing CRC and reducing CRC-related deaths across the population is screening average-risk individuals [5]. Therefore, most European countries, Canada, specific regions in North and South America, Asia, and Oceania have initiated population-based screening programs [6]. Eligibility to participate in CRC screening is determined by age and area of residence. The results of microsimulation modeling have shown a downward trend in CRC morbidity and mortality in the United States, attributed to the implementation of screening programs [5]. In addition, population-based screening aims to reveal latent disease among the average-risk population, enabling early-stage interventions and reducing the threat to individuals and/or communities [7].

Screening is particularly appropriate in CRC, as it is not only a common disorder but one thought to be characterized by a gradual development of the adenoma–carcinoma sequence [8]. Although the time taken for an early adenoma to progress to an established CRC is yet unknown, the current evidence suggests it to be no less than ten years [9], offering abundant opportunity for detection via screening followed by treatment. Furthermore, CRC can be prevented by removing colorectal adenomas [10], and the earlier the CRC is detected, the less likely the patient will die [11]. Treatment outcomes are thus positively impacted by interventions along the adenoma–carcinoma pathway. Other effective strategies include identifying and monitoring high-risk populations, including individuals with inflammatory bowel disease, families with hereditary CRC syndrome, individuals whose family history suggests a genetic predisposition to CRC but have no detectable genetic markers, and individuals whose phenotypic appearance indicates high risk. The metabolome helped identify the important biological activities affected by genetic variation [12]. The most frequently used CRC screening methods are fecal occult blood tests (FOBTs) and lower endoscopy [13].

CRC development takes years. Generally, 10 to 15 years is required for a polyp to form a malignant tumor. So, regular screening, detecting, and removing polyps at the early stage is crucial; thereby, CRC can be prevented. Current diagnosis can detect only 40% of CRC cases in the early stages, and CRC might recur following surgery and post-surgery treatment [14]. Chemotherapeutic medications target cancer cells while also harming healthy cells in the environment. Modern chemotherapies acquired resistance in nearly all CRC patients, decreasing anticancer drug efficacy and ultimately leading to chemotherapy failure. Therefore, a comprehensive discussion on the epidemiology, the risk factors, and preventive measurement of CRC based on the updated evidence-based knowledge is crucial to overcome the future challenges of CRC. This review discussed the current global epidemiology, drug resistance, its challenges, risk factors, and preventive and treatment strategies of CRC. Additionally, there is a brief discussion on CRC carcinogenesis and recommendations for preventing and treating CRC.

## 2. Colorectal Cancer Development

CRC is genetically diverse; however, it can develop through various unique mechanisms. For example, many CRC cells exhibited dozens of somaclonal mutations resulting from the distinct level of gene expression profiles. As a result, CRC is believed to have one of the most incredible mutational loads of any malignancy. Based on the number of somaclonal mutations, CRC can be broadly categorized as hypermutated (more than 12 mutations per 10^6^ bases) or non-hypermutated (fewer than 8.24 mutations per 10^6^ bases) [15]. In addition, a novel categorization system for CRC was developed as a result of parallel efforts to categorize CRC based on gene expression profiles. These classifications have been updated and modified due to integrating data on gene expression profiles with tumor genotypes [15].

CRC develops when epithelial cells acquire a series of genetic or epigenetic changes that enable them to be hyperproliferative [16]. These rapidly developing cells form a benign adenoma, which can advance to cancer and metastasize via several distinct pathways, including microsatellite instability (MSI), chromosomal instability (CIN), and serrated neoplasia [17,18,19]. Adenoma–carcinoma sequence is a term used to describe cancer progression. The traditional pathway is responsible for the vast majority of sporadic CRC cases. Cancer begins as a tiny adenoma that becomes a giant adenoma and, finally, cancer. There is a strong association between this pathway and the development of the chromosomal instability (CIN)-positive subtype (CIN-positive). According to the National Cancer Institute, this model accounts for 10–15% of sporadic CRC. It is defined by development from normal cells to hyperplastic polyp, sessile serrated adenomas, and eventually cancer [13]. This route is typically involved in developing the CpG island methylator phenotype (CIMP)-high subtype, the pathway involved in inflammation. The prolonged inflammation causes normal cells to develop indeterminate dysplasia, which progresses further to low-grade dysplasia, progresses further to high-grade dysplasia, and finally, cancer [13]. Inflammatory bowel illnesses and the widespread use of prophylactic colectomy are responsible for less than 2% of CRC cases worldwide. There are benign precursor lesions accessible and removed in all pathways, albeit more noticeable in the adenoma–carcinoma and serrated pathways [13]. Because they take years to grow into cancer, there is a window of opportunity for secondary prevention of colorectal cancer.

**Figure 1:**
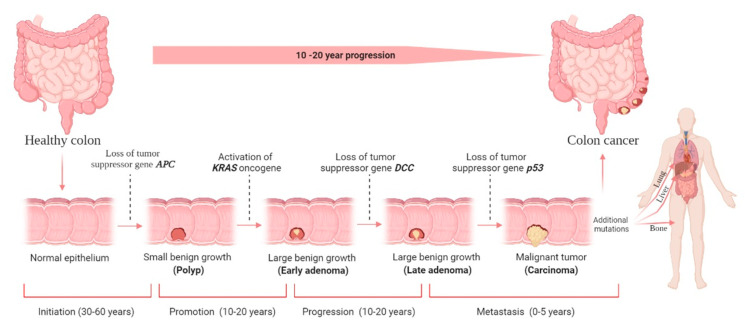
Colorectal cancer (CRC) stages and development. There are four stages in the development of CRC carcinogenesis: initiation, promotion, progression, and metastasis. The liver is the most common metastatic site, followed by the lung and bone. Although it is difficult to determine the duration required for each stage, decades will likely be required to form CRC. The figure was created using BioRender.com (accessed on 30 December 2021).

When an adenocarcinoma becomes invasive, it can spread to other body parts via blood and lymphatic arteries (Figure 1). Adenocarcinomas account for roughly 96% of all CRC [20]. However, up to 18 years may pass between developing a polyp and invasive cancer. On average, it takes nine years to form metastasis [21]. Like any other tumor or cancer, CRC is classified by stage 0 (carcinoma in situ) through stage IV (Figure 1).

Usually, a non-cancerous development results in dysplastic tissue formation (tumor), leading to CRC development once the cells have undergone several abnormal DNA changes. A non-cancerous (benign) soft tissue tumor is a growth that does not spread (metastasize) to other parts of the body. Hyperproliferation causes a (benign) polyp or adenoma to form (stage 0). Ten percent of adenomatous polyps can become malignant, forming an adenocarcinoma that invades the muscularis propria (stage I). The tumor grows in volume and further invades tissue in the serosa (stage II) and visceral peritoneum (stage III). Then, there is the possibility of developing lymphatic or blood vessel metastasis (stage IV) [22]. The stage determines the severity of the disease and the therapy options available [23]. Even though surgery is the standard treatment choice for stages 0–II CRC, stage III CRC requires surgery and adjuvant chemotherapy, and stage IV and recurrent CRC require surgery, chemotherapy, and targeted therapy; however, unfortunately, it has no known certainty established cure until now.

## 3. Current Global Epidemiology of Colorectal Cancer

**Figure 2:**
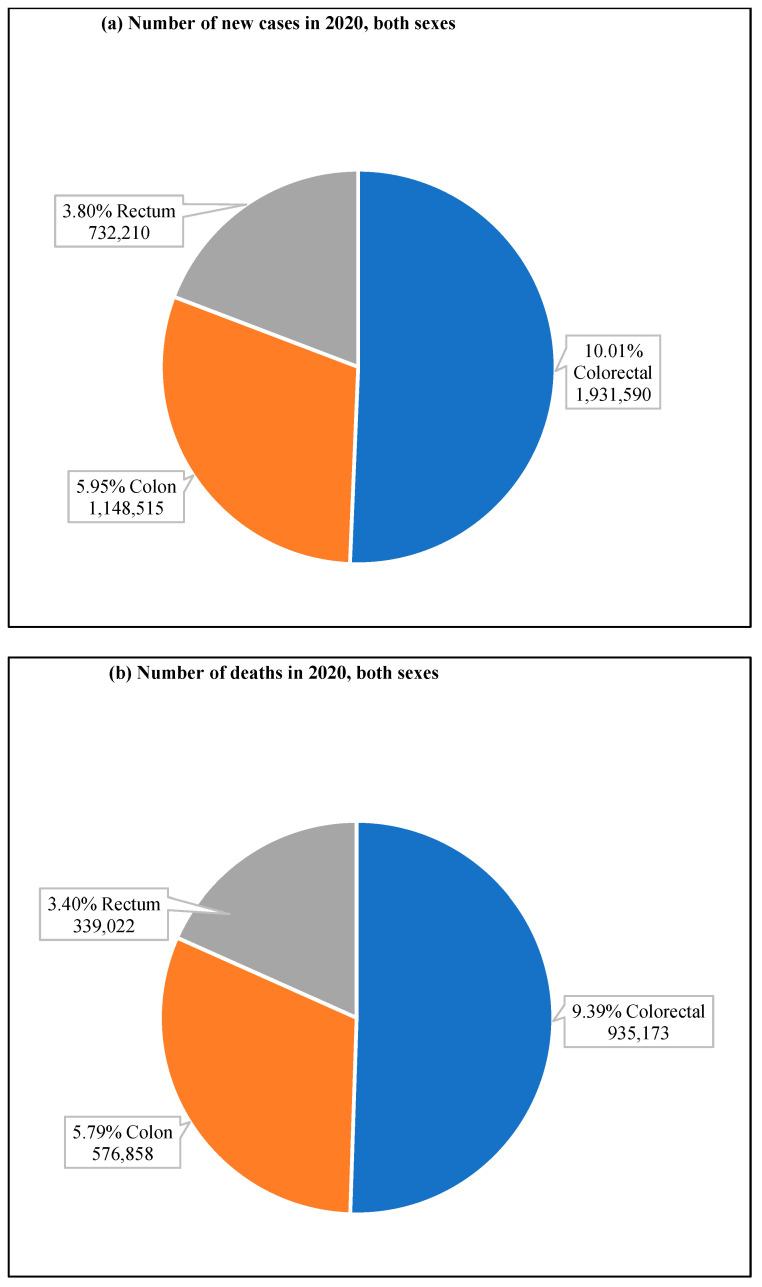
CRC new cases and deaths in 2020. (**a**) shows new cases, both sexes and all ages, and (**b**) shows deaths of both sexes for all age groups. The value shown in % is calculated against the total number of all cancers. The data source is GLOBOCAN [25], taken with permission.

**Figure 3:**
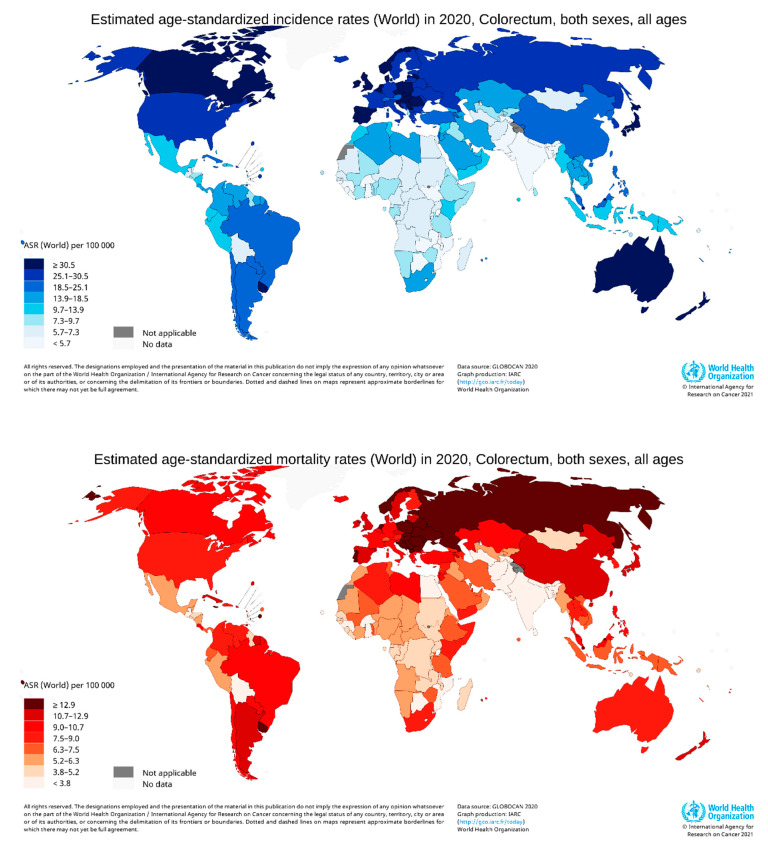
Map showing the global distribution of estimated age-standardized incidence rates (**top**) and mortality rate (**bottom**) of CRC in 2020 for both sexes and all ages. (Reproduced from GLOBOCAN [25] with permission).

According to GLOBOCAN data, in 2020, there were an estimated 19.3 million new cases and 10 million cancer deaths worldwide, of which CRC contributed about 1.93 million (10%) further incidences and 0.94 million (9.4%) deaths (Figure 2a,b). The overall estimated age-standardized global distribution of incidence and mortality of CRC for both sexes and all ages is shown in Figure 3. The incidence and mortality of CRC vary considerably between countries and among world regions. They are also associated with the socioeconomic status of the country. According to the World Bank, the new cases and deaths are more remarkable in areas with higher income levels and lesser in areas with lower income levels.

**Figure 4:**
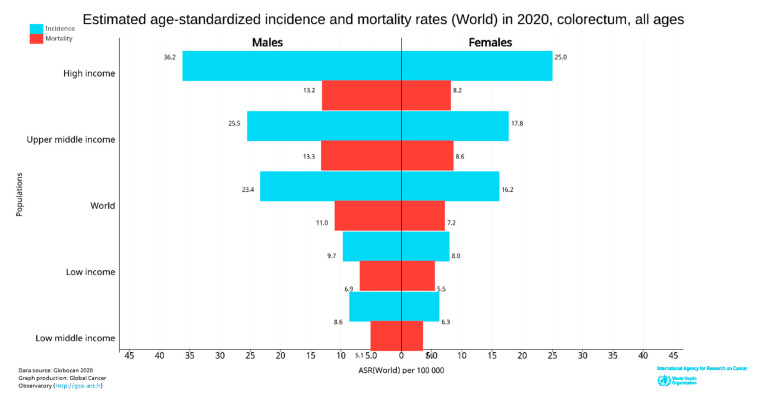
World CRC incidence and mortality rates in 2020 (according to income level for all age groups). The data were extracted from GLOBOCAN [25] with permission.

The incidence and deaths of CRC are increasing in developing countries for both males and females, even though it is significantly high in high-income countries. The upper-middle-income countries accounted for the highest incidences (45.94%) and deaths (49.37%). The higher-income countries recorded fewer incidences (42.43%) than upper-middle-income countries; however, the deaths were significantly lower (36.40%), perhaps due to better treatment facilities (Figure 4). The high-income and upper-middle-income countries covered more than 88 and 85% of incidence and mortality, respectively (Table 1) [24].

In 2020, CRC was the most diagnosed cancer (out of 36 cancers) among men in 18 of the 186 countries worldwide and women in 6 of the 185 countries [24]. However, in 2018, CRC was the most diagnosed among men in 10 of 185 countries, and no country had CRC as the most diagnosed cancer among women [26]. So, the CRC incidence rate has increased to about 10 from 5% in the last two years in men. Women were predominant in 3.24% of countries (Figure 4). CRC is more common among men than women and more than four times more common in high-income countries than low-income countries. The deaths were also about 2.5 times higher in high-income nations than in low-income nations.

In 2020, age-standardized (world) incidence rates per 100,000 of CRC in both sexes were 19.8, with males having a higher incidence rate of 23.4 and females with 16.2, which is almost equal to the incidence of 2018 [24,26]. The death rate in both sexes was 9.1; for men was 11, and for women was 7.2 per 100,000 CRC (Figure 4).

**Figure 5:**
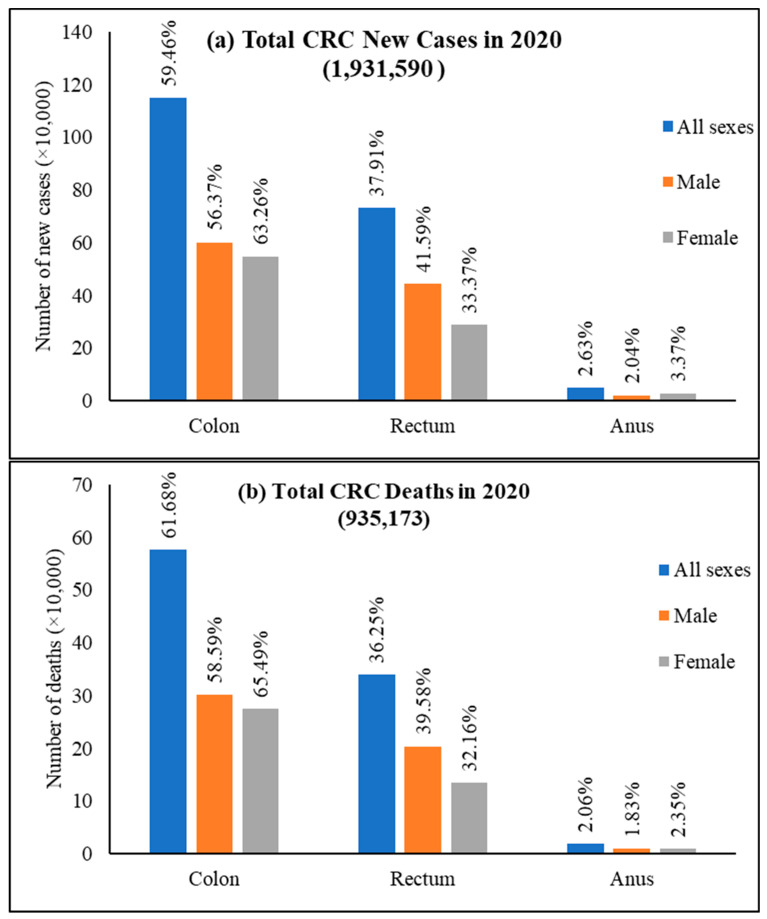
World new cases and deaths of colon cancer, rectum cancer, and anus cancer in 2020. (**a**) New cases and (**b**) deaths of colon cancer, rectum cancer, and anus cancer. The value is shown in % on top of each column and is calculated against the CRC number in 2020 for both sexes and all ages. Data extracted from GLOBOCAN [25] with permission.

Among CRC, colon cancer was predominant and accounted for 59.5% of new cases, 61.9% of deaths and rectum cancer had 37.9% of incidence and 36.3% mortality for both sexes and all ages (Figure 5). Colon cancer alone stands fifth for new cancer cases and deaths compared to all cancers. In contrast, rectum cancer is the eighth and tenth most severe cancer for incidences and mortality, respectively [24].

## 4. Cancer Drug Resistance and Its Challenges

Germline abnormalities due to the mutation of adenomatous polyposis coli (APC) or DNA mismatch repair or K-ras or p53 genes lead to the uncontrolled proliferation of cells and progression to CRC, often correlating with specific stages of the developmental process [27,28,29]. The mutated genes produce the mutated protein that cannot respond typically, and damaged DNA continues the proliferation and accumulates further genetic mutations leading to a malignant phenotype (Leslie et al., 2002). These consequences are not understandable at the early stage of CRC. Therefore, early detection of CRC is complex, and its limitation causes a high death rate. Apart from the early detection problem, there are myriad issues in CRC diagnosis. The key diagnostic hurdles include pre-operative staging and imaging methods that can accurately detect lymph node illness and/or micro-metastatic disease. This would affect patient care significantly.

Surgery is a standard treatment procedure for CRC, particularly for stage 0 to stage II [30,31]. Other stages required adjuvant therapy or chemotherapy and targeted therapy besides the surgery. However, one of the most significant issues in surgery is determining the function of laparoscopic resection in patients with a shallow level of rectum cancer. In both circumstances, CRC is frequent, and procedures that can be widely used must be developed. This has ramifications for both techniques and training. Postoperative care and prognostication in patients with CRC are greatly aided by pathological evaluation of tumor resection material. In addition, there are issues in examining and reporting these specimens, either because of difficulty in implementing existing criteria or introducing new concepts [32]. Patients with CRC require high-quality pathology reports to receive an accurate prognosis and guidance for patient management.

Drug resistance against chemotherapeutic regimes imposes another challenge to surviving the increasing number of CRC patients. In recent decades, the overall survival of individuals with advanced colon cancer has increased due to new chemotherapy regimens. Although the response rate to modern systemic chemotherapies can reach as high as 50%, drug resistance is reported to develop in nearly all patients with CRC, limiting the therapeutic efficacy of anticancer drugs and ultimately leading to chemotherapy failure [33,34].

## 5. Risk Factors of Colorectal Cancer

The development of CRC is related to non-modifiable risk factors and modifiable risk factors. Personal medical history (sex, age, race, the history of adenomatous polyps, inflammatory bowel disease (IBD) history) and family history cannot be controlled by individuals. The modifiable factors are related to individual habits or lifestyles. By modifying or altering the modifiable factors, the risk for CRC can be reduced.

### 5.1. Personal and Medical History

Young adults and teenagers can develop colorectal cancer; however, most colorectal malignancies occur over 50 years. Men are on average 68 years old when diagnosed with colon cancer, while women are on average 72 years old. Both men and women are diagnosed with rectal cancer at 63 years on average [35,36]. Increasing age is one of the non-modifiable risk factors of CRC. A study conducted by Steele et al. [36] revealed that of the 7948 CRC patients, 77% were between 50 and 79 years of age.

Although both genders are likely to develop CRC, males are more likely than females to develop CRC [37]. In a recent comparative study, compared with controls, patients with early-onset CRC (18–49 years) were more likely to be male (odds ratio [OR], 1.87; CI95% 1.39–2.51), have IBD (3 vs. 0.4% for controls; univariable *p* < 0.01). Furthermore, compared with late-onset CRC (50 years or older), patients with early-onset CRC were more likely to be male (OR, 1.44; CI95% 1.11–1.87), black (OR, 1.73; CI95% 1.08–2.65) or Asian (OR, 2.60; CI95% 1.57–4.15), and have IBD (OR, 2.97; CI95% 1.16–6.63) [38]. Importantly, related to the anatomic site of CRC, males had significantly higher odds relative to females for rectal cancer (OR 2.84, CI95% 2.25–3.58) than distal cancer (OR 1.84, 95% CI 1.50–2.24) [39].

Based on the CDC report, in 2020, CRC incidence and mortality differ by sex, race, and ethnicity. Overall, black men and women had the highest incidence and mortality rates among races, followed by white men and women, Asian or Pacific Islanders, and American Indians or Alaska Natives. Men and women of non-Hispanic origin have a higher incidence and mortality rate than Hispanic men and women. The incidence of late-stage colorectal cancer was 30 to 60% lower in several Asian American groups (Korean and South Asian men and women and Chinese women) [40,41]. Relative to white people, black people had significantly lower rectal cancer with OR 0.88 but increased distal with OR 1.27 and proximal with OR 1.62 [39]. African Americans were more likely to have tumors with OR 1.78 and more likely to have proximal tumors than white people with OR 4.37 [37,42].

CRC is linked to IBD, such as ulcerative colitis or Crohn’s disease. This mechanism is probably due to chronic mucosal inflammation, increased cell turnover, and increased rates of sporadic mutations [43]. In a meta-analysis of 13 trials involving nearly 45,000 patients with IBD, the risk of CRC was shown to be approximately three times greater in people with IBD (RR 2.93, 95% CI 1.79–4.81) than in people with no IBD [44]. A retrospective study revealed that compared to control, patients with CRC have IBD (3% for IBD vs. 0.4% for the control group, *p*-value < 0.01) [38].

Epidemiological studies have shown that individuals with type 2 diabetes mellitus have an increased risk of developing CRC. A case-control study in Africa revealed that diabetes mellitus was independently associated with colorectal cancer with OR 5.3, 95% CI: 1.4–19.9, *p* = 0.012. Furthermore, a study with nearly 22,000 CRC patients in the US showed that diabetes prevalence was more strongly associated with proximal (OR 1.29, 95% CI: 1.22–1.36) than distal (OR 1.15, 95% CI: 1.08–1.22) or rectal cancer (OR 1.12, 95% CI: 1.06–1.19) [39,45]. The effect of hyperinsulinemia on the colon is one underlying mechanism that could explain an increased risk of proximal colon cancer. Insulin-like growth factors (IGFs), which function in growth and development and are overexpressed in cancer cells together with specific IGF receptors, play a role in developing many malignancies. IGF does so by increasing cell cycle progression while also suppressing apoptosis. Insulin promotes cancer by stimulating insulin receptors and lowering the amounts of IGF-binding proteins in the body. As a result, the amount of free unbound IGF increases. The Ras–Raf–MEK–Mitogen-Activated Protein Kinase (MAPK) pathway and the phosphatidylinositol 3-kinase (PI3K)–AKT– mammalian target of rapamycin (mTOR) pathway are both activated as a result of this activity. Both of these proteins are involved in cell development and proliferation. More significant insulin signaling leads to increased metabolic activity within the cell, which leads to more oxidative stress and DNA damage [46,47].

### 5.2. Family History

Family history is usually defined as a first-degree relative with CRC. A family history of cancer was associated with an increased risk for CRC. A large-scale meta-analysis involving 8091 cases of CRC in 16 studies concluded that the mean risk of CRC was almost two times higher in individuals with a family history of CRC compared to those with no family history of CRC (RR 1.80, 95% CI: 1.61 to 2.02) [44]. A retrospective study in the US about risk factors associated with early-onset CRC found that patients with early-onset CRC (18–49 years old) with a family history of CRC have a higher risk of developing CRC compared to patients with no family history of CRC (OR 8.61, CI 4.83–15.75). Meanwhile, compared to patients with late-onset CRC (patients ≥50 years old), patients with early-onset CRC were more likely to have a family history of CRC with OR 2.87, 95% CI: 1.89–4.25 [38]. Furthermore, a study about the site-specific risk for CRC in the Korean population revealed that a family history of cancer was associated with a higher risk of distal colon cancer in both men and women and proximal colon cancer in men [48].

Familial adenomatous polyposis (FAP) and Lynch syndrome, also known as hereditary nonpolyposis colorectal cancer (HNPCC), are the two most common forms of hereditary colon cancer. Both conditions result from certain germline mutations that contribute to an increased risk of CRC among family members. FAP is a rare genetic disease caused by the APC gene mutation that inhibits tumor formation in the gut. Hundreds manifest the diseases to thousands of small, adenomatous polyps lining the large intestine and rectum, usually during adolescence. Polyps persist proliferate throughout the colon, with eventual transformation into malignancy. The risk of developing colorectal cancer for individuals with untreated FAP is almost 100% [49,50].

Lynch syndrome is the most frequent hereditary propensity for CRC and is an autosomal dominant inherited illness. Lynch syndrome is caused by germline mutations in one of the DNA mismatch repair (MMR) genes, including MLH1, MSH2, MSH6, or PMS2, or epithelial cell adhesion molecule (EpCAM). For carriers of germline MMR mutations, the lifetime risk of acquiring colorectal cancer is predicted to be over 60%. Colorectal cancer affects multiple generations in a family, and it develops early in life, with a mean age at diagnosis of roughly 45 years [49,50,51].

### 5.3. Lifestyle and Dietary Intake

Obesity is associated with an elevated risk of CRC. A meta-analysis study that examined the relationship between CRC with body mass index (BMI) involving more than 66,000 CRC patients in 23 studies found a significant association between BMI and CRC. The risk of CRC increased by ten percent every increasing BMI of 8 kg/m^2^ [44]. Another meta-analysis of prospective studies revealed that increasing BMI and waist circumcision (per 10-cm increase) were associated with an increased risk of colon cancer in both men and women. The relative risk was higher in men. BMI was significantly correlated with rectal cancer in men but not in women [52]. The mechanism underlying the relationship between obesity-linked CRC risk is that obesity promotes insulin resistance or hyperinsulinemia, chronic inflammation, oxidative stress, DNA damage, and elevated insulin-like growth factor-1 (IGF-1) levels, stimulating cell proliferation [47].

Physical inactivity is associated with an elevated risk of CRC. On the other hand, high levels of physical activity increased the survival rate in CRC patients. A meta-analysis of 52 studies found that physical exercise frequency and intensity are inversely related to CRC risk [53].

Regular alcohol consumption, both weekly or daily, is significantly associated with an increased risk of CRC [35]. An individual with moderate and heavy (≥4 drinks/day) alcohol consumption has a 21 and 52% risk of CRC, respectively [54]. There was a time-dependent relationship between the duration of alcohol consumption with CRC. The longer the period of alcohol consumption, the higher the risk of having CRC [55]. After more than 14 years of follow-up, a Danish population-based cohort study revealed that drinkers of more than 40 drinks a week had a relative risk of rectal cancer of more than twice compared to non-drinkers [56]. A Korean cohort study involving more than 3000 men and more than 1000 women showed that moderate and heavy alcohol consumption was associated with an increased risk for distal colon cancer in men and a higher risk for rectal cancer in women [48]. Alcohol metabolism involves the conversion of ethanol to its metabolites which can cause carcinogenic effects in the colon. The production of ethanol metabolites can be influenced by the colon microbiota, another recently recognized mediating factor for colon carcinogenesis. The development of DNA adducts, oxidative stress and lipid peroxidation, epigenetic changes, epithelial barrier dysfunction, and immunological modulatory effects are all linked to the production of acetaldehyde and alcohol’s other metabolites. Furthermore, alcoholics are susceptible to a poor diet deficient in folate and fiber, as well as circadian disruption, which may exacerbate alcohol-induced colon cancer [57].

Cigarette smoking is a modifiable risk factor for developing CRC, and the risk of CRC increases with the increasing number of cigarettes consumed [35,38,44]. Compared to non-smokers, the relative risks of CRC were 1.06 for 5 pack-years, 1.11 for 10 pack-years, 1.21 for 20 pack-years, and 1.26 for 30 pack-years [44]. Smoking is also linked to a decreased CRC-specific survival rate, especially among current smokers [49]. Compared to people who never smoked, current smoking was more strongly associated with rectal cancer (OR 1.81, 95% CI: 1.68–1.95) than proximal cancer (OR 1.53, 95% CI: 1.43–1.65) or distal cancer (OR 1.46, 95% CI: 1.35–1.57) [39]. Furthermore, male smokers had a 39 percent higher risk of distal cancer. In comparison, female ever smokers had a 20 percent higher risk of proximal cancer when compared to never smokers of the same gender. Female smokers had a greater risk of rectal cancer compared to male smokers [58]. One of the carcinogens found in cigarettes is nicotine. Nicotine has increased proliferation by altering receptor expression and phosphorylation patterns in various mitogenic pathways. Nicotine exposure leads to higher phosphorylation of epidermal growth factor receptor (EGFR) and increased expression of 5-LOX in colon cancer. Similarly, nicotine increases the growth of colorectal cancer cells by upregulating acetylcholine and noradrenaline receptors. Nicotine also stimulates angiogenesis and neovascularization in colon cancer through increases in VEGF, 5-LOX, COX-2, and matrix metalloproteinase-2/9 (MMP-2/9) [59].

Based on the accumulated evidence from prospective epidemiological studies and meta-analysis studies, the consumption of red (beef, pork, lamb) and processed meat raises CRC risk by 20–30% [49,60]. Another meta-analysis study showed that consumption of red meat for five servings/week has a 13% higher risk of CRC [44]. A cohort study involving more than 4000 patients reported that frequent meat consumption was linked to an increased incidence of proximal colon cancer in males and rectal cancer in women [44]. A prospective study in the UK found that individuals who reported consuming an average of 76 g/day of red and processed meat had a 20% higher risk of CRC than 21 g/day [61]. It is recommended that healthy people consume red meat a maximum of 500 g/week or 70 g/day, and there is a limitation to consuming processed meat [60].

On the other hand, white meat such as fish and poultry is safe and not associated with CRC risk [60,61]. The mechanism behind the association between excessive red and processed meat consumption and CRC risk is unknown. However, the potential underlying mechanisms for this association include carcinogenic substances formed during the cooking process that may increase exposure to N-nitroso compounds (NOCs), heterocyclic amines (HCAs), and polycyclic aromatic hydrocarbons (PAHs). Heme iron molecules in red meat can produce carcinogens as well as act as DNA mutagens themselves. PUFAs, bile acids, non-human sialic acid, and infectious pathogens are all potential mechanistic drivers as well [43,60]. To reduce the carcinogenic effects of HCAs, a diet rich in dietary fiber sources such as wheat bran and vegetables is recommended. Avoiding exposure of meat surfaces to flames, using aluminum foil to wrap meat before oven roasting, and microwave cooking can help decrease the formation of HCAs [60].

On the other hand, consuming high-fiber dietary patterns such as vegetables, fruits, whole grains, and cereals is associated with a low incidence of CRC. The prospective study with more than 2600 cases of CRC reported that the highest fifth of fiber intake from bread and breakfast cereals was associated with a 14% (95% CI: 2–24) decreased risk of CRC [61]. In addition, the American Cancer Society and the World Cancer Research Fund recommend a diet high in vegetables, fruits, and whole grains to prevent cancer [49]. Furthermore, vitamin D deficiency is associated with CRC [62]. Therefore, supplemental calcium and vitamin D indicate a potential decreased risk of CRC [63,64]. The recommended dose of calcium supplementation is 700–1250 mg/day, and recommended daily dose of vitamin D for the high-risk population of CRC [65].

### 5.4. Medication and Hormones

Regular (at least two doses/week) aspirin and other nonsteroidal anti-inflammatory drugs (NSAIDs) are associated as protective factors of CRC. A meta-analysis study involving 1967 colon cancer patients and 6684 CRC patients reported that the relative risk of CRC with five years of aspirin or NSAID was 0.60 in case-control studies and 0.80 in cohort studies [44]. Similar to the case-control study with more than 21,000 CRC patients revealed that aspirin use was significantly more strongly associated with reduced rectal cancer (OR 0.71) than distal (OR 0.85) or proximal (OR 0.91) [39]. Using aspirin and NSAIDs in the long term risks major gastrointestinal bleeding or heart attack from selective COX-2 inhibitors. Because of these side effects, the American Cancer Association does not recommend aspirin or NSAIDs in the general population. Daily use of low-dose aspirin is recommended to prevent cardiovascular disease and CRC for certain individuals in their 50s who are at high risk for cardiovascular disease and should be made after discussion with health workers [49]. The recommended dose of aspirin for CRC prevention for the high-risk population is 80–1200 mg/day. In contrast, NSAIDs for CRC prevention is not recommended in either the general population or the high-risk population due to the toxicity profile of NSAIDs [65].

Exogenous postmenopausal hormone replacement therapy is associated with a decreased risk of CRC, cancer-related mortality, and all-cause mortality [65,66]. A meta-analysis study revealed that hormonal therapy (HT) is stronger in present HT users than in former HT users. After ten years, current users had a 39% lower risk than previous users, who had a 16% lower risk. The relative risks of CRC were 0.89 (95% CI: 0.73–1.09) for 1 year, 0.80 (95% CI: 0.54–1.17) for 2 years, 0.65 (95% CI: 0.26–1.68) for 5 years, and 0.61 (95% CI: 0.10–3.96) for 10 years of HT use compared to no HT use [44]. A study in Sweden with more than 1000 CRC patients reported that HT use after CRC diagnosis was associated with a 26% risk reduction in CRC mortality with a hazard ratio (HR) of 0.67 and a 30% risk reduction in all-cause mortality with an HR 0.68 [66]. A cohort study in Norway found that the current use of HT was associated with a lower risk of CRC with a relative risk was 0.88 compared with no use of HT. Furthermore, the relative risk of current use of estrogen therapy was 0.91, while the relative risk for current use of combined estrogen-progestin therapy was 0.85 [67].

## 6. Preventive Strategies

Significant ground-breaking knowledge of molecular mechanisms behind CRC development revealed that dietary factors might be associated with CRC development at an increasing rate. However, the evidence to date is regrettably inadequate due to its highly complex mechanisms. However, another significant association of intestinal microbiota with CRC has been predicted. Again, intestinal microbiota balance depends on the dietary habits and alterations of balanced intestinal microbiota involved with CRC development and progression [68,69]. However, modulation of the gut microbiota is a promising strategy to enhance treatment efficacy and reduce the adverse effects of CRC therapies [68]. Many challenging issues, including etiology, diagnosis, treatment, and management, need to be addressed to properly manage CRC and identify the key concerns for a long-term solution.

### 6.1. Primary Prevention

Primary prevention strategies are used in an otherwise healthy population to prevent CRC. It is anticipated that if exposure to risk factors continues to decline at its current pace, the recent downward trend in CRC incidence and mortality in the US will persist. If the pace of risk factor modification increases, the incidence rates could fall even further. Risk factor modification is possible for many cancers, although modification generally causes a decrease in incidence rates over a long rather than a short time horizon; hence, considerable time is required to observe and assess how changes in the prevalence of CRC risk factors impact incidence rates [70].

Epidemiological research has identified several potentially modifiable factors, presenting both challenges and opportunities related to primary prevention. CRC is possible to be prevented by reducing cigarette smoking [71], excessive alcohol consumption [72], overweight or obesity [73], and the consumption of large amounts of red and processed meat [74]. Equally, it has been established that the risk of CRC can be reduced by physical activity [75], regular aspirin consumption [76,77], and hormone replacement therapy [78], while certain studies have also found that consumption of milk and whole grains may help protect against CRC [74].

### 6.2. Secondary Prevention

Unlike other cancers, CRC is generally characterized by slow development over several years to decades after normal colorectal epithelium is transformed into an adenoma [79]. As the adenoma–carcinoma sequence evolves so slowly, secondary prevention of CRCs is frequently possible via colonoscopy, when adenomas can be detected and removed—detecting CRC at an earlier stage. At the same time, still treatable enables more effective secondary prevention of deaths. The most frequently employed screening methods are endoscopic examinations of the large bowel (particularly flexible sigmoidoscopy and colonoscopy) and stool tests such as FOBTs. Although capsule endoscopy, computed tomography (CT), other stool-based (e.g., DNA-based) tests, and blood or urine tests have been suggested, the take-up of these methods has been small due to doubts over their diagnostic performance and their high cost. CT, moreover, is known to have side effects. However, researchers in many countries seek novel biomarkers, such as blood-based ‘omics signatures’, which could bring critical new advancements to the current range of available noninvasive or minimally invasive CRC screening tests [80].

### 6.3. Tertiary Prevention

There is mounting epidemiological evidence that several of the important risk factors for developing CRC also impact survival outcomes, suggesting multiple opportunities for tertiary prevention. For example, despite the recognized difficulty of persuading individuals to change unhealthy lifestyle factors, it is thought that a cancer diagnosis offers a valuable ‘teachable moment’ that can be harnessed to urge patients to make necessary lifestyle modifications.

Two important modifiable risk factors for CRC are cigarette smoking and heavy alcohol consumption. Both appear to be linked to lower survival rates [81,82,83,84], highlighting the urgent need to encourage and assist patients to achieve necessary lifestyle changes. Although the exact mechanism associating lower survival rates with these specific risk factors has not been identified, higher rates of surgical complications, suboptimal responses to chemotherapy and radiotherapy, and nicotine-induced suppression of cancer cell apoptosis and enhancement of cell migration are all thought to play a role [85,86].

In contrast, evidence demonstrates a relationship between physical activity and positive cancer outcomes, including less fatigue, improved quality of life, and more prolonged survival [87,88,89]. Among the mechanisms investigated and proposed to explain this link are the reduced whole-body and visceral fatness, metabolic dysregulation, chronic inflammation, oxidative stress, and the enhanced immune function associated with participation in physical activity [90]. Further development of this body of research requires large-scale randomized controlled trials (RCTs) assessing the short- and long-term effects of different types of physical activity during and after the postsurgical period. RCTs already carried out have shown a positive association between outcomes and certain physical activity-based interventions, indicating that cancer treatment and monitoring should be accompanied by short- and long-term physical activity programs that are individually designed to meet each patient’s needs and condition. A level of physical activity of 17.5 to 35 metabolic equivalent task (MET) hours per week is strongly recommended for CRC patients, which has cut mortality by 30 to 40%. Patients with limited physical capabilities should engage in at least 3.5 MET-hours per week of physical activity [91]. A study involving 573 women with stage I and III CRC revealed that increasing levels of exercise after a diagnosis of non-metastatic CRC reduced cancer-specific mortality with *p* for trend was 0.008, and overall mortality with *p* for trend was 0.003 [92]. However, a prospective cohort study that included more than 1200 patients found no statistically significant association of physical activity with overall survival among patients with metastatic CRC [93].

Significant research is currently being undertaken on the possibility of using chemoprevention in tertiary prevention. Multiple observational studies have concluded that the consumption of low-dose aspirin, which is already associated with reduced CRC risk, also promotes survival rates after CRC diagnosis [94], possibly due to COX inhibition [95]; investigations of additional roles of non-COX mechanisms have been initiated to explore and define a potential role for aspirin in tertiary prevention [96]. RCTs are also required to evaluate further initial indications that metformin can enhance survival rates among patients with CRC [97]. However, it is thought that significant flaws, such as immortal time bias, in certain important pharmacoepidemiological studies [98] led to the frequent administration of certain drugs, including beta-blockers, to treat comorbidities in patients with CRC, which have since been demonstrated to have no value in this population.

Epidemiological research has also indicated an association between vitamin D deficiency, commonly seen in patients with CRC [62], and significantly lower chances of survival [99,100]. Among multiple mechanisms proposed to explain this association is the immunomodulatory, antiangiogenetic, and proapoptotic effects of vitamin D. A systematic review and meta-analysis of randomized controlled trials reported that vitamin D supplementation has a clinically significant effect on CRC survival outcomes [101,102]. The findings of a recent randomized phase II trial indicated improved progression-free survival among patients with metastatic CRC who took high-dose vitamin D supplements [103]. Further RCTs are required to assess the role of vitamin D supplementation in tertiary prevention [104].

## 7. Treatment Strategies

For many years, cancer patients have been treated with surgery and chemotherapy as the initial lines of defense against the disease. However, individuals with metastatic disease have historically had a poor prognosis for CRC. Primary and adjuvant therapy advancements have improved CRC survival time. In usual cases, surgery is required to remove the tumor [105] altogether. Nearly a quarter of CRC cases are diagnosed at the advanced stage, and 20% of the remaining cases acquire metachronous metastases; therefore, curative surgical control alone is often challenging, resulting in tumor-related mortality [13]. Notably, chemotherapy or radiotherapy may be used before or after surgery to help shrink or stabilize the tumor [105]. Current chemotherapy comprises single-agent therapy (primarily fluoropyrimidine (5-FU)) and multiple-agent regimens, including oxaliplatin (OX), irinotecan (IRI), and capecitabine (CAP or XELODA or XEL). The combined therapy regimens FOLFOX (5-FU + OX), FOXFIRI (5-FU + IRI), XELOX or CAPOX (CAP + OX), and CAPIRI (CAP + OX) remain the mainstream approaches in first-line treatment. Patients with poor performance or low risk of deterioration are recommended single-agent therapy. Choosing additive agents appears to be similar in efficacy, with only side effects varying [106]. However, it has several irreversible drawbacks, such as systemic toxicity, unsatisfactory response rates, variable innate and acquired resistance, and low tumor-specific selectivity. As a result, a large amount of money has been invested in developing innovative ways to refine or even replace standard CRC chemotherapy. The following paragraphs present a discussion on different novel approaches, including immunotherapy and gene therapy for the management of colorectal cancer.

### 7.1. Targeted Therapy

Targeted therapy is a revolutionary novel strategy demonstrated to considerably enhance overall survival in people with the disease when it comes to CRC. Because of the increasing effectiveness of treatments such as the anti-EGFR (epidermal growth factor receptor) agent cetuximab and the anti-angiogenesis agent bevacizumab are innovative medications that inhibit numerous immunological pathways checkpoints are being developed at an unprecedented rate [106]. There are multiple checkpoints discovered, including immune checkpoint (T cell), vascular endothelial growth factor/vascular endothelial growth factor receptor (VEGF/VEGFR), epidermal growth factor/epidermal growth factor receptor (EGF/EGFR), hepatocyte growth factor (HGF), mesenchymal-epithelial transition factor (c-MET), insulin-like growth factor/insulin-like growth factor 1 receptor (IGF/IGF-1R), transforming growth factor (TGF), Wnt/β-catenin, Notch, and Hedgehog that can be potentially targeted for the CRC treatment [106]. The United States of America Food and Drug Administration (USA-FDA) approved several targeted agents for CRC to target these checkpoints. The first targeted agent for CRC is Cetuximab which was approved in 2004 by FDA. After this, several are now approved (Table 2). In addition, there have been numerous new FDA-approved targeted medicines for CRC that have been released to the market since then, with more on the way (Table 2). Multiple preclinical and clinical studies have been undertaken on the various drugs developed [106].

Nanotechnology is a rapidly growing field in drug delivery that offers several advantages over traditional methods. Colon-specific innovative delivery mechanisms would enable the local distribution of a high concentration of medications in the colon, which would enhance pharmacotherapy while reducing systemic toxicity and other adverse outcomes. Theranostic nanocarriers have been created to monitor and treat illness while utilizing a single delivery system [107,108,109,110,111,112,113,114,115,116,117].

### 7.2. Gene Therapy

It entails using genetic components to address a variety of illnesses, including cancer. The genetic component may be a nucleic acid such as DNA or RNA, which can help substitute or repair malfunctioning genes. Gene therapy can also be employed to trigger an immunological response or as a treatment in and of itself.

Mutation and gene aberration play a role in colorectal cancer progression. The potential to inhibit CRC may lie in modifying and correcting these faulty genes and preventing overexpressed genes. The transformation of many genes has a role in colon cancer progression. Cancer can be caused by point mutations, the creation of oncogenes, the deregulation or deletion of proto-oncogenes, and the loss of activity of suppressor-oncogenes [118].

As of November 2017, nearly 2600 clinical studies had been undertaken in 38 countries, of which more than half included only phase I trials [119]. Only 45 of the 1309 gene therapy studies reached phase III. In the United Kingdom, 11 gene treatments for CRC are currently being tested [120]. Approximately 50,000 to 100,000 genes in the human body and only a handful participate in the cell cycle. Faulty genes may be one of the most important causes of CRC, and it has been estimated that defective genes cause at least 30% of colon cancers. Some of them have been linked to colon cancer in families. The main advantage of gene therapy is the transfer of particular genes to specific tumor cells, which suppresses the aberrant function of the mutant gene and slows tumor progression [118,121,122,123].

### 7.3. Immunotherapy

Tumor immunotherapy has piqued the interest of researchers since it shows great therapeutic promise in CRC. There are several ongoing clinical studies on immunotherapies in humans with CRC. Monoclonal antibody (mAb) therapy, immune checkpoint inhibitors therapy, cancer vaccines, adoptive cell therapy, complement inhibition, and cytokine treatment are some immunotherapy techniques used in CRC [124]. Most of these are in phase I or II, with encouraging outcomes in several research studies. Over 24 immunotherapy-based clinical studies for human CRC have been undertaken to date, with more than 40 trials currently enrolling or about to enlist patients [125,126,127,128,129,130,131,132].

#### 7.3.1. Monoclonal Antibody Therapy

In this therapy, humanized antibodies such as Cetuximab and Panitumumab, which preferentially identify the epidermal growth factor receptor (EGFR), are used to treat metastatic CRC. Currently, there are clinical studies ongoing on some MAbs for CRC, such as adecatumumab against epithelial cell adhesion molecule (EpCAM), labetuzumab against carcinoembryonic antigen (CEA), and pemtumomab against Mucins [133].

#### 7.3.2. Immune Checkpoint Inhibitors Therapy

CTLA-4, an immunological checkpoint molecule that binds to CD80/CD86 structures on antigen-presenting cells, inhibits T-cell activation (APC). The T-cell function is negatively regulated by programmed death receptor ligand 1/2 (PD-L1/L2), which binds to the PD-1 receptor on T-cells and is typically stimulated by their various ligands, which are expressed on either tumor cells (e.g., PDL1/L2PD-1) or APCs (e.g., CD80/86 CTLA-4; PD-L1/L2PD-1). Activated CTLA-4 and PD-1 immune checkpoint signal. For CRC, a phase II clinical study of the single medication Nivolumab and a mix of dual therapies such as Nivolumab + Ipilimumab is now underway [134].

#### 7.3.3. Cancer Vaccines

Cancer vaccines have been developed to stimulate antigen-specific T- or B-cell activity against cancer by delivering antigens to antigen-presenting cells (APCs) such as dendritic cells (DCs). Vaccines also contain components designed to activate DCs that have been pulsated with antigens and direct them to a local lymph node, for example, the DC vaccination and OncoVAX [135].

##### DC Vaccine

Since most CRCs express the tumor-associated carcinoembryonic antigen (CEA), DCs can be pulsed with CEA mRNA or CEA peptides. Most CRC people who underwent DC vaccination exhibited CEA-specific T-cell immunological responses in advanced CRC patients [136].

##### OncoVAX

This was created to employ patients’ cancer cells combined with an immune-stimulating adjuvant to elicit antitumor immune activity and prevent colon cancer relapse following surgery. An amalgamation of targeted immunotherapy and surgery significantly increases patient survival rates [137,138].

### 7.4. Adoptive T-Cell Therapy

This treatment can enhance antitumor immunity and vaccine effectiveness. Recent studies have focused on imbuing effector T-cells with desirable antigen receptors, such as chimeric antigen receptor T-cells. In a human colon cancer xenografted mice model, ex vivo-grown human Vδ1 γδ T cells showed extraordinary therapeutic potential [139].

### 7.5. Complement Inhibition

Complement is an integral part of the immune system. The activation of complement is a vital aspect of the immune surveillance reaction to CRC. Complement is an element of the innate and adaptive immune systems and consists of approximately 30 fragments and proteins. In a mouse colon cancer model, complement inhibitors such as cobra venom factor, humanized cobra venom factor, and recombinant Staphylococcus aureus superantigen, such as protein 7, were tested. Complement depletion is an effective type of immunotherapy for CRC as it can slow tumor progression by increasing the host’s immune responses to cancer and decreasing the immunosuppressive effect of the tumor microenvironment. It can also be used as a combination immunotherapy regimen [140].

### 7.6. Cytokine Therapy

Cytokines are important components of tumor immunology, notably in CRC, where the inflammatory process and immunogenic responses drive tumor development. TNF and interleukin-6 are critical factors in CRC as they stimulate the major oncogenic factors, nuclear factor-B, and inducer of transcription 3 (STAT3) in intestinal cells, promoting proliferation and resistance against apoptosis [141].

### 7.7. Natural Products as Therapy

Nature contains plenty of sources for the first line of treatment and prominent compounds to treat many severe diseases [142,143]. An estimation reported that approximately 75–80% of the world’s population uses traditional medical systems as their first therapy option due to lack of access to treatment facilities as well as worries about synthetic medicine safety and efficacy [144,145]. On the other hand, natural products have grown in importance as sources of polypharmacological treatments for infectious diseases, malignancies, and neurological disorders [146,147]. Moreover, references to plants in religious texts have prompted more scholars to examine the scientific validity of traditional claims [148,149,150]. Therefore, natural products are an excellent source of drugs that can be possible treatment opportunities for CRC.

Ongoing research and development into natural products result in nearly 50% of currently available cancer treatments being derived directly or indirectly from natural ingredients [151,152]. These natural products can be of different types, including alkaloids, polysaccharides, polyphenols, diterpenoids, and unsaturated fatty acids, with various structural properties, each with its unique set of characteristics. The discovery of new natural products has ushered in a new era in cancer prevention and treatment. The natural product-based medications currently being tested in clinical trials for CRC treatment have been critically reviewed recently by Huang et al. [151]. There are many phytocompounds in different phases of clinical trials for CRC treatment, including andrographolide (phase II), berberine (phase II/III), curcumin (phase I), epigallocatechin gallate (phase II), metformin (phase II), methotrexate (phase II), resveratrol (phase I), silymarin (phase IV), SN-38 (phase II), and topotecan (phase II). These compounds have the potential to exert anti-CRC effects by interfering with the pathways of metastasis, invasion, apoptosis, and angiogenesis, all of which are major hallmarks of human cancer [153,154,155,156,157].

Natural products have been shown to provide undeniable benefits; therefore, these could be used to treat CRC as well as other cancers. However, drug resistance and adverse effects are still critical issues in CRC treatment. Some natural compounds have been coupled with classical chemotherapeutic medicines, such as 5-FU, to increase the cancer cells’ susceptibility to conventional therapy, resulting in a new technique for treating CRC [151]. A network meta-analysis demonstrates that Ai-Di, Shen-Qi-Fu-Zheng, and Matrine injections, which the Chinese FDA has approved, improve the overall response rate and quality of life in advanced colorectal cancer patients treated with oxaliplatin, 5-fluorouracil, and leucovorin, as well as reduce peripheral neurotoxicity (III-IV) [158]. The strategy increases potency while reducing side effects.

Consumption of nutritionally and medicinally important herbs and spices could be another safer preventive measure for CRC. When used in large dosages, herbs and spices used in cooking, which have been used for centuries, have been found to have a CRC protective impact or even to be helpful as an anti-CRC adjuvant therapy when used in conjunction with chemotherapy [159,160,161]. Herbs and spices include a wide variety of bioactive chemicals, which have a variety of beneficial health impacts. The primary mechanisms herbs and spices exert are the BCL-2, K-ras, and MMP pathways, caspase activation, the extrinsic apoptotic pathway, and the modulation of ER-stress-induced apoptosis chemopreventive effects [162,163].

## 8. Recommendations

The incidence of CRC will likely increase in the coming decades due to population growth and aging and the increased prevalence of important lifestyle risk factors, including physical inactivity, overweight, obesity, and poor diet. Hence, a higher number of both cases and deaths are anticipated without effective prevention programs. Primary prevention is a long-term process whose outcomes can only be fully assessed and appreciated well into the future. Nonetheless, given the considerable overlap between the risk and protective factors of CRC and those of other common chronic diseases, both other common cancers and cardiovascular and metabolic diseases, there could be a beneficial spillover from primary prevention initiatives targeted at reducing CRC risk factors. Consequently, such initiatives should be given high priority within cancer control programs. Meanwhile, cost-effective and efficient secondary prevention, such as fecal immunochemical testing, flexible sigmoidoscopy, and colonoscopy, can effectively decrease the CRC burden. Even over a ten- to the twenty-year horizon, significant reductions in CRC mortality could be achieved if these screening tools were implemented in effectively organized, high-quality, widescale programs with high adherence rates. CRC screening tools are not only cost-effective themselves but should be regarded as a means of ensuring further long-term cost savings. Moreover, it can be anticipated that future screening strategy will elicit additional cost benefits, for example, when novel molecular markers—particularly blood-based omics signatures—are identified and developed as noninvasive or minimally invasive tests. Furthermore, new evidence indicates that effective CRC tertiary prevention strategies, such as prescribing aspirin, discouraging cigarette smoking, and encouraging physical activity, have further potential to improve survival and quality of life among patients with CRC.

Several procedures must be taken to manage CRC properly. Aside from early detection and screening of high-risk communities and individuals, maintaining a healthy diet and lifestyle is essential. This study recommends that culinary herbs and spices help to prevent and treat CRC by inhibiting the growth of human colorectal cancer cells via molecular signaling pathways. In addition to public cancer registration, the government should provide quality medical care for timely diagnosis and treatment, better-personalized therapy, and easy access to clinical trials for CRC patients, and raise awareness of CRC and other comorbidities to improve cancer care and research.

## 9. Conclusions

There is an urgent need to tackle the alarming rate of CRC effectively. It will take coordinated efforts to eliminate modifiable risk factors, leverage chemoprevention research, and promote population-wide and targeted screening to achieve the most significant possible reduction in CRC incidence and mortality. As multiple risk factors contribute to CRC development, creating awareness among the general population is crucial. At the same time, healthy foods, especially fruits, culinary spices, and herbs, need to be included in the daily meal. Therefore, the effort committed to each strategy must be matched with the overall health priorities of the specific population, taking into consideration fiscal resources and healthcare infrastructure. In addition, evaluation of PDENs widely as targeted drug delivery to the cancer cell lines is highly recommended for further study.

## Figures and Tables

**Table 1:** World CRC estimated age-standardized incidence and mortality rates in 2020 (all ages).

Population *	Incidence (%)	Mortality (%)
Upper middle income	887,025 (45.94)	461,511 (49.37)
High income	819,143 (42.43)	340,272 (36.40)
Low middle income	194,954 (10.10)	112,556 (12.04)
Low income	29,542 (1.53)	20,392 (2.18)
Total	1,930,664	934,731

* Data extracted from GLOBOCAN [25] with permission.

**Table 2:** The USA-FDA approved immunotherapy drugs for colorectal cancer.

Immunotherapy Drugs	Receptor	Type of Protein	Year of Approved
Cetuximab	EGFR	Transmembrane protein	2004
Bevacizumab	EGFR, VEGF/VEGFR	Transmembrane/signaling protein	2004
Panitumumab	EGFR, VEGF/VEGFR	Transmembrane/signaling protein	2006
Ziv-aflibercept	VEGF/VEGFR	Signaling protein	2012
Regorafenib	VEGF/VEGFR	Signaling protein	2012
Ramucirumab	VEGF/VEGFR	Signaling protein	2015
Pembrolizumab	Block PD-1	Programmed cell death protein 1	2017
Nivolumab	Block PD-1	Programmed cell death protein 1	2017
Ipilimumab	Block PD-1	Programmed cell death protein 1	2018

VEGF: vascular endothelial growth factor, VEGFR: vascular endothelial growth factor receptor, EGFR: epidermal growth factor receptor.
